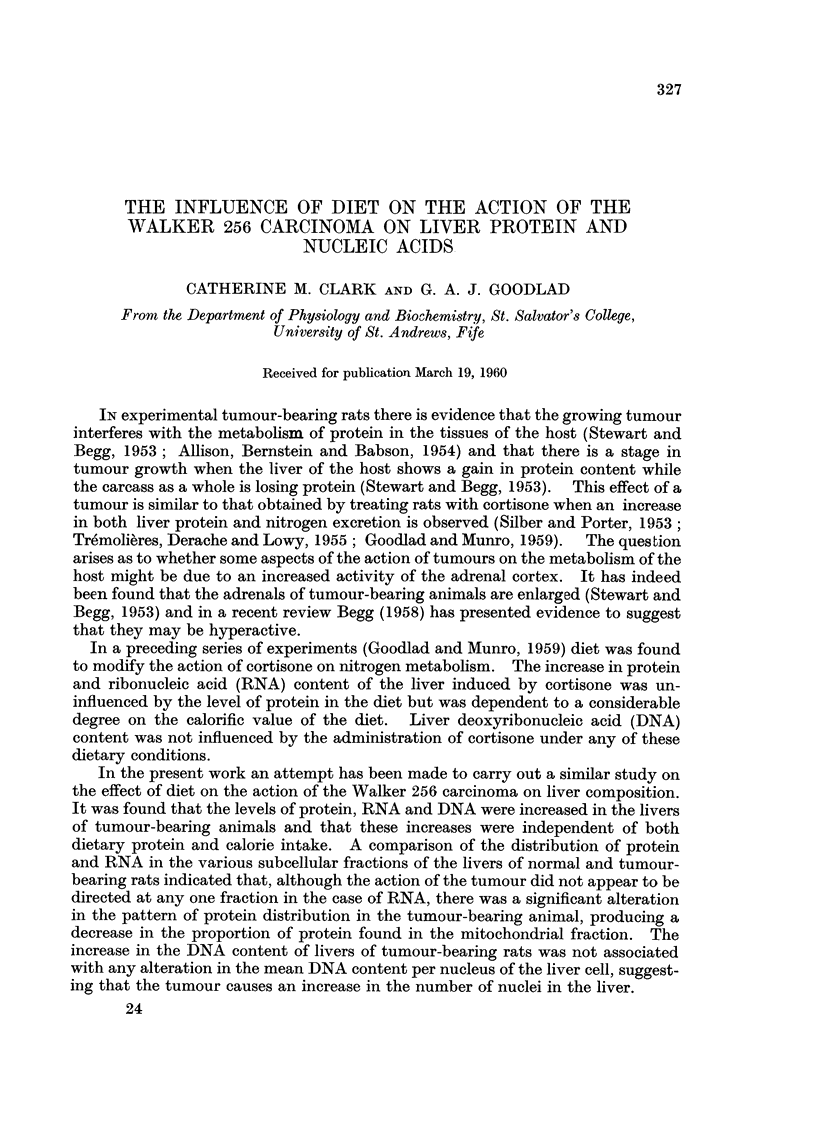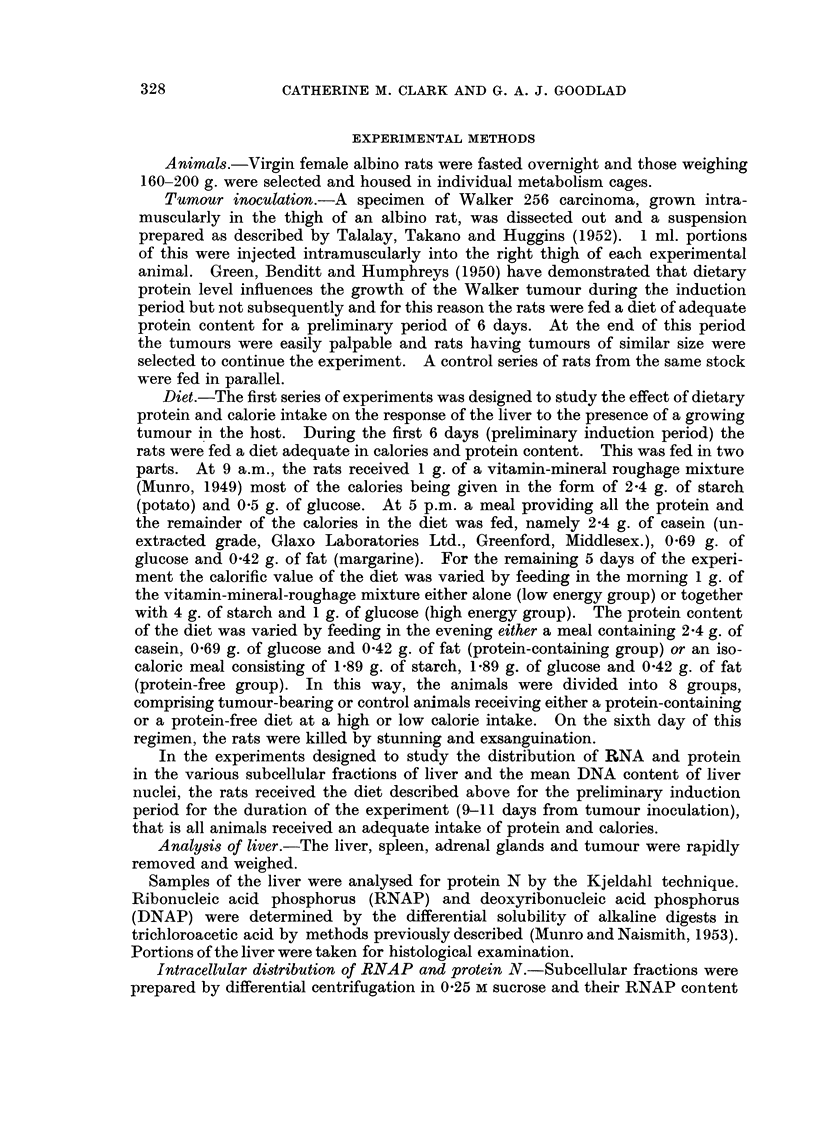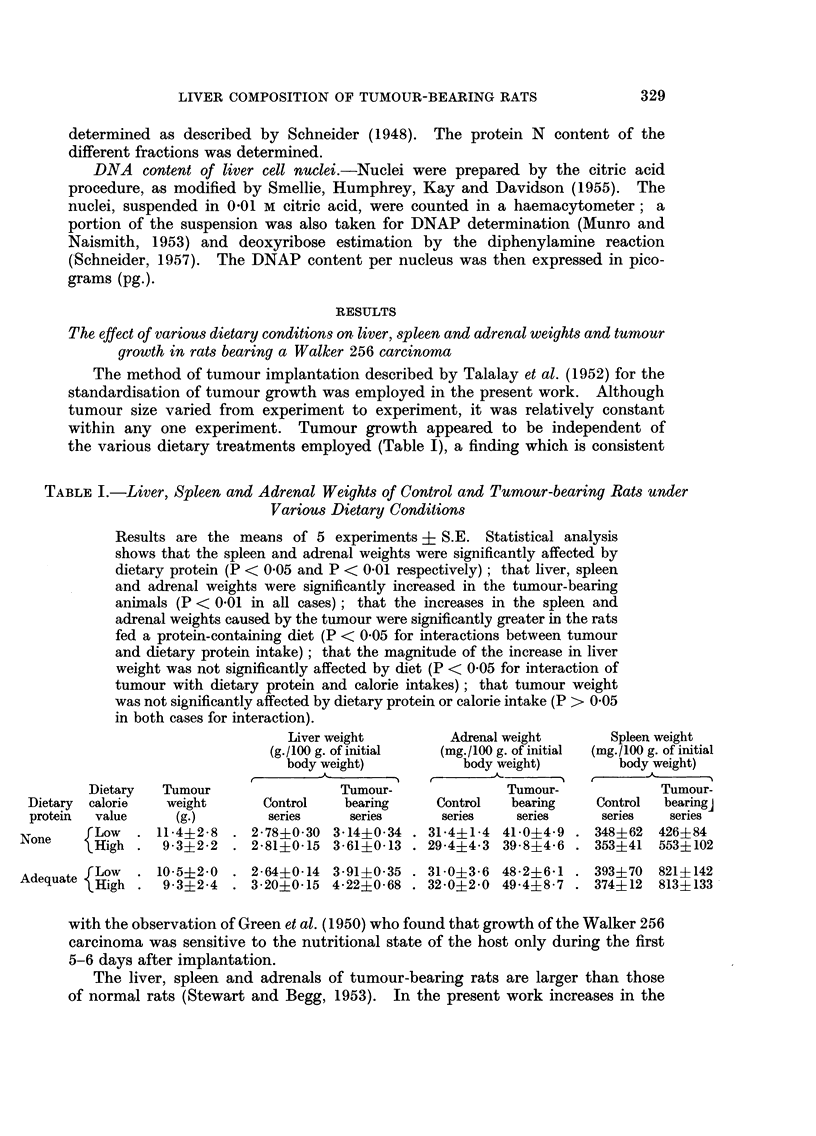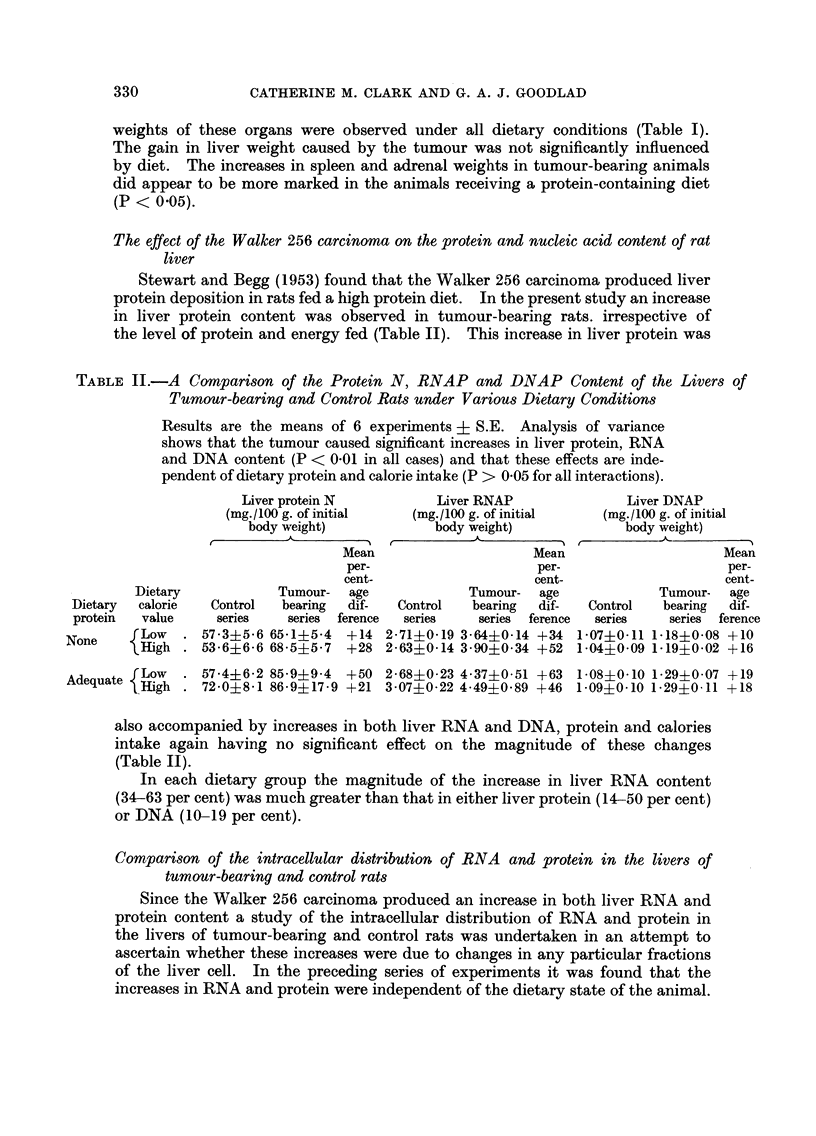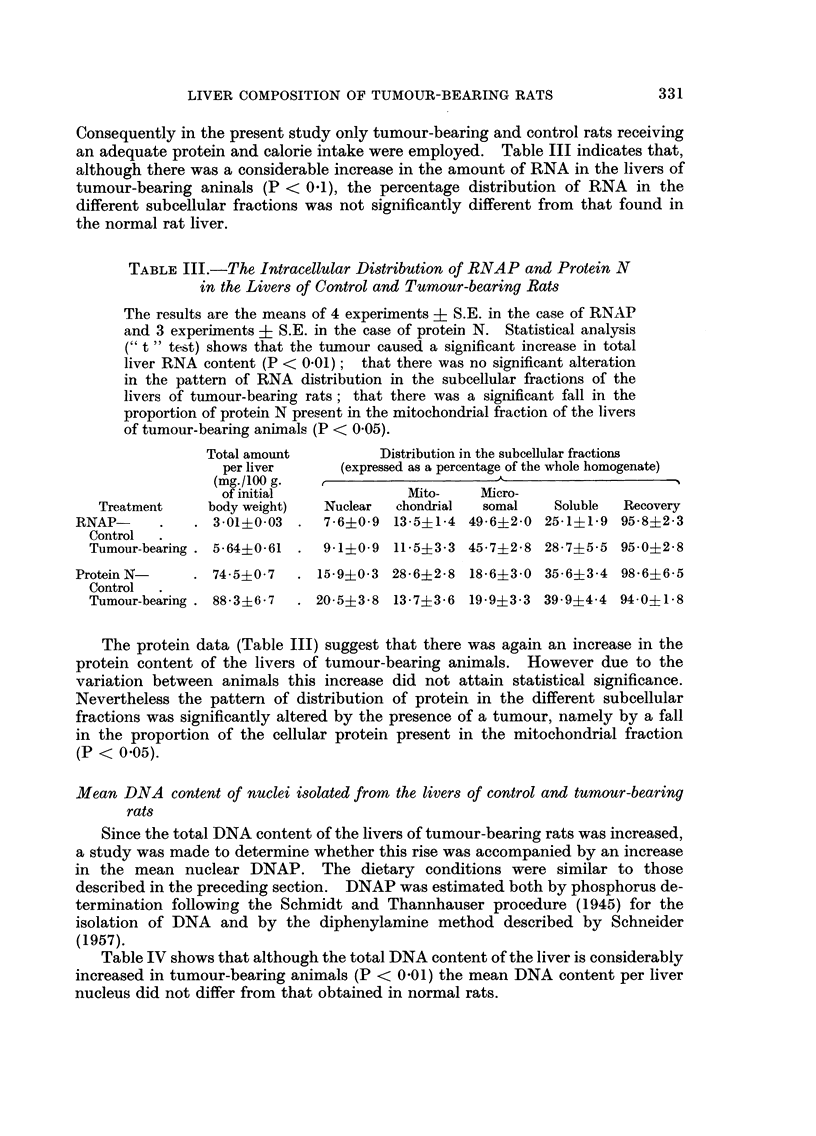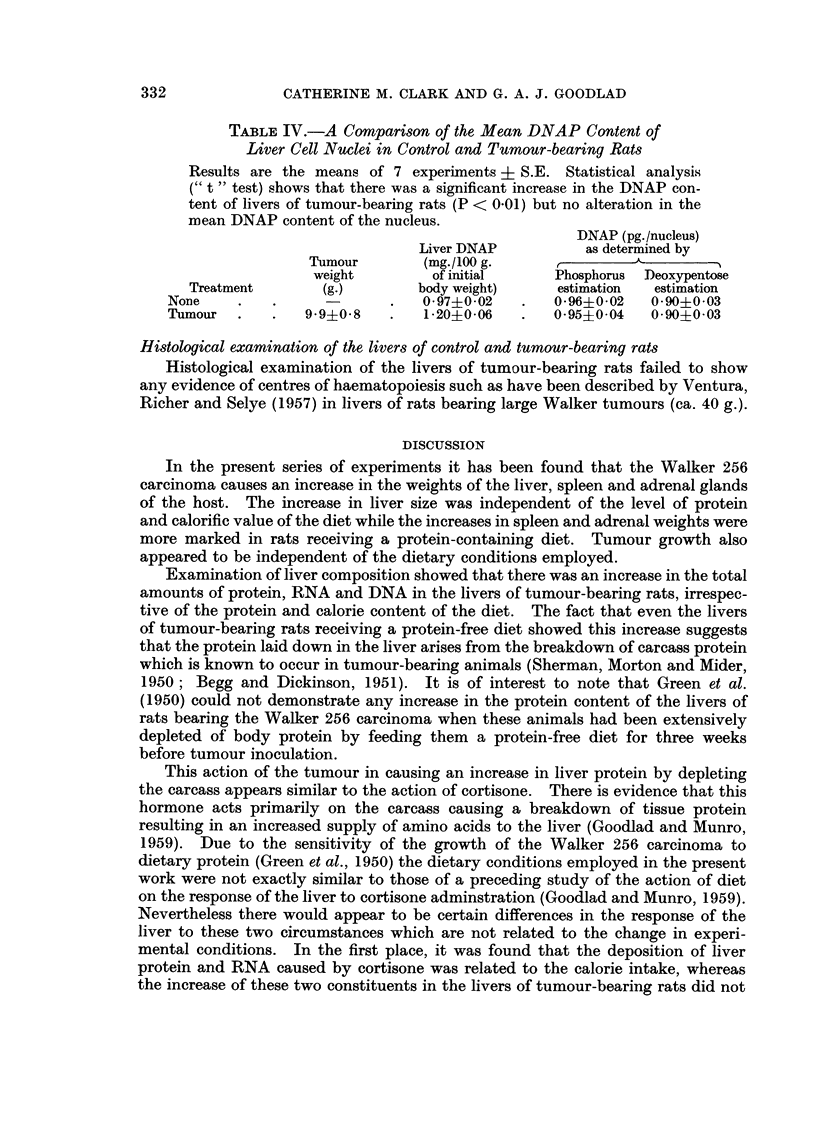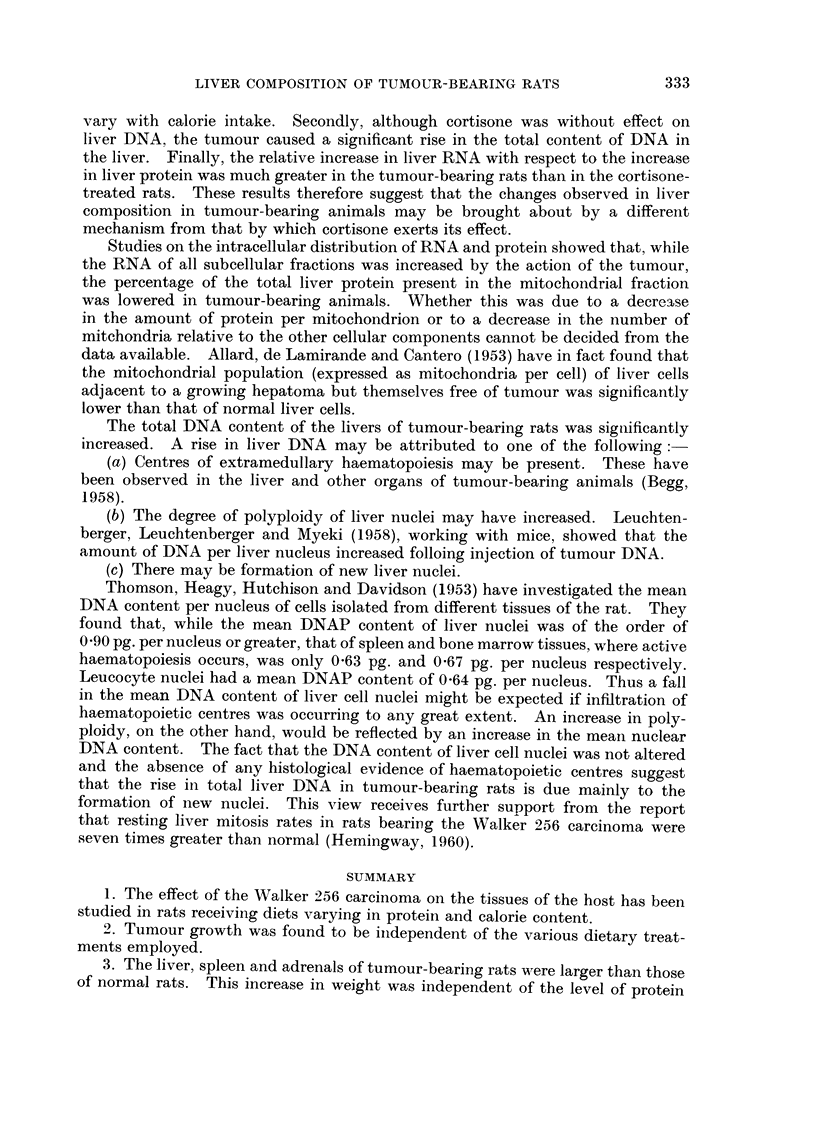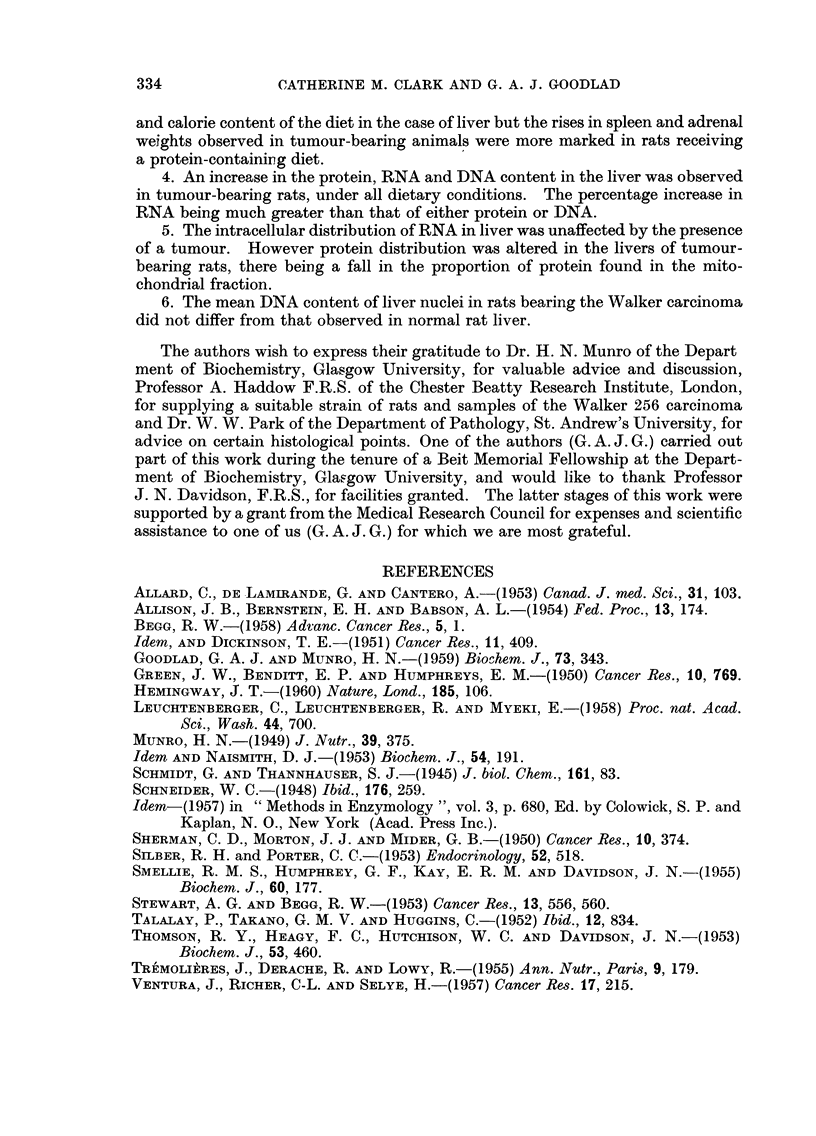# The Influence of Diet on the Action of the Walker 256 Carcinoma on Liver Protein and Nucleic Acids

**DOI:** 10.1038/bjc.1960.39

**Published:** 1960-06

**Authors:** Catherine M. Clark, G. A. J. Goodlad


### THE INFLUENCE OF DIET ON THE ACTION OF THE WALKER 256 CARCINOMA ON LIVER PROTEIN AND NUCLEIC ACIDS

CATHERINE M. CLARK AND G. A. J. GOODLAD

*From the Department of Physiology and Biochemistry, St. Salvator's College, University of St. Andrews, Fife*

Received for publication March 19, 1960

IN experimental tumour-bearing rats there is evidence that the growing tumour interferes with the metabolism of protein in the tissues of the host (Stewart and Begg, 1953 ; Allison, Bernstein and Babson, 1954) and that there is a stage in tumour growth when the liver of the host shows a gain in protein content while the carcass as a whole is losing protein (Stewart and Begg, 1953). This effect of a tumour is similar to that obtained by treating rats with cortisone when an increase in both liver protein and nitrogen excretion is observed (Silber and Porter, 1953 ; Trémolières, Derache and Lowy, 1955 ; Goodlad and Munro, 1959). The question arises as to whether some aspects of the action of tumours on the metabolism of the host might be due to an increased activity of the adrenal cortex. It has indeed been found that the adrenals of tumour-bearing animals are enlarged (Stewart and Begg, 1953) and in a recent review Begg (1958) has presented evidence to suggest that they may be hyperactive.

In a preceding series of experiments (Goodlad and Munro, 1959) diet was found to modify the action of cortisone on nitrogen metabolism. The increase in protein and ribonucleic acid (RNA) content of the liver induced by cortisone was uninfluenced by the level of protein in the diet but was dependent to a considerable degree on the calorific value of the diet. Liver deoxyribonucleic acid (DNA) content was not influenced by the administration of cortisone under any of these dietary conditions.

In the present work an attempt has been made to carry out a similar study on the effect of diet on the action of the Walker 256 carcinoma on liver composition. It was found that the levels of protein, RNA and DNA were increased in the livers of tumour-bearing animals and that these increases were independent of both dietary protein and calorie intake. A comparison of the distribution of protein and RNA in the various subcellular fractions of the livers of normal and tumour-bearing rats indicated that, although the action of the tumour did not appear to be directed at any one fraction in the case of RNA, there was a significant alteration in the pattern of protein distribution in the tumour-bearing animal, producing a decrease in the proportion of protein found in the mitochondrial fraction. The increase in the DNA content of livers of tumour-bearing rats was not associated with any alteration in the mean DNA content per nucleus of the liver cell, suggesting that the tumour causes an increase in the number of nuclei in the liver.

### EXPERIMENTAL METHODS

*Animals.*—Virgin female albino rats were fasted overnight and those weighing 160–200 g. were selected and housed in individual metabolism cages.

*Tumour inoculation.*—A specimen of Walker 256 carcinoma, grown intramuscularly in the thigh of an albino rat, was dissected out and a suspension prepared as described by Talalay, Takano and Huggins (1952). 1 ml. portions of this were injected intramuscularly into the right thigh of each experimental animal. Green, Benditt and Humphreys (1950) have demonstrated that dietary protein level influences the growth of the Walker tumour during the induction period but not subsequently and for this reason the rats were fed a diet of adequate protein content for a preliminary period of 6 days. At the end of this period the tumours were easily palpable and rats having tumours of similar size were selected to continue the experiment. A control series of rats from the same stock were fed in parallel.

*Diet.*—The first series of experiments was designed to study the effect of dietary protein and calorie intake on the response of the liver to the presence of a growing tumour in the host. During the first 6 days (preliminary induction period) the rats were fed a diet adequate in calories and protein content. This was fed in two parts. At 9 a.m., the rats received 1 g. of a vitamin-mineral roughage mixture (Munro, 1949) most of the calories being given in the form of 2·4 g. of starch (potato) and 0·5 g. of glucose. At 5 p.m. a meal providing all the protein and the remainder of the calories in the diet was fed, namely 2·4 g. of casein (unextracted grade, Glaxo Laboratories Ltd., Greenford, Middlesex.), 0·69 g. of glucose and 0·42 g. of fat (margarine). For the remaining 5 days of the experiment the calorific value of the diet was varied by feeding in the morning 1 g. of the vitamin-mineral-roughage mixture either alone (low energy group) or together with 4 g. of starch and 1 g. of glucose (high energy group). The protein content of the diet was varied by feeding in the evening *either* a meal containing 2·4 g. of casein, 0·69 g. of glucose and 0·42 g. of fat (protein-containing group) *or* an isocaloric meal consisting of 1·89 g. of starch, 1·89 g. of glucose and 0·42 g. of fat (protein-free group). In this way, the animals were divided into 8 groups, comprising tumour-bearing or control animals receiving either a protein-containing or a protein-free diet at a high or low calorie intake. On the sixth day of this regimen, the rats were killed by stunning and exsanguination.

In the experiments designed to study the distribution of RNA and protein in the various subcellular fractions of liver and the mean DNA content of liver nuclei, the rats received the diet described above for the preliminary induction period for the duration of the experiment (9–11 days from tumour inoculation), that is all animals received an adequate intake of protein and calories.

*Analysis of liver.*—The liver, spleen, adrenal glands and tumour were rapidly removed and weighed.

Samples of the liver were analysed for protein N by the Kjeldahl technique. Ribonucleic acid phosphorus (RNAP) and deoxyribonucleic acid phosphorus (DNAP) were determined by the differential solubility of alkaline digests in trichloroacetic acid by methods previously described (Munro and Naismith, 1953). Portions of the liver were taken for histological examination.

*Intracellular distribution of RNAP and protein N.*—Subcellular fractions were prepared by differential centrifugation in 0·25 M sucrose and their RNAP content determined as described by Schneider (1948). The protein N content of the different fractions was determined.

*DNA content of liver cell nuclei.*—Nuclei were prepared by the citric acid procedure, as modified by Smellie, Humphrey, Kay and Davidson (1955). The nuclei, suspended in 0·01 M citric acid, were counted in a haemacytometer; a portion of the suspension was also taken for DNAP determination (Munro and Naismith, 1953) and deoxyribose estimation by the diphenylamine reaction (Schneider, 1957). The DNAP content per nucleus was then expressed in picograms (pg.).

### RESULTS

*The effect of various dietary conditions on liver, spleen and adrenal weights and tumour growth in rats bearing a Walker 256 carcinoma*

The method of tumour implantation described by Talalay *et al.* (1952) for the standardisation of tumour growth was employed in the present work. Although tumour size varied from experiment to experiment, it was relatively constant within any one experiment. Tumour growth appeared to be independent of the various dietary treatments employed (Table I), a finding which is consistent

TABLE I.—*Liver, Spleen and Adrenal Weights of Control and Tumour-bearing Rats under Various Dietary Conditions*

Results are the means of 5 experiments ± S.E. Statistical analysis shows that the spleen and adrenal weights were significantly affected by dietary protein ($P < 0{\cdot}05$ and $P < 0{\cdot}01$ respectively); that liver, spleen and adrenal weights were significantly increased in the tumour-bearing animals ($P < 0{\cdot}01$ in all cases); that the increases in the spleen and adrenal weights caused by the tumour were significantly greater in the rats fed a protein-containing diet ($P < 0{\cdot}05$ for interactions between tumour and dietary protein intake); that the magnitude of the increase in liver weight was not significantly affected by diet ($P < 0{\cdot}05$ for interaction of tumour with dietary protein and calorie intakes); that tumour weight was not significantly affected by dietary protein or calorie intake ($P > 0{\cdot}05$ in both cases for interaction).

| Dietary protein | Dietary calorie value | Tumour weight (g.) | Liver weight (g./100 g. of initial body weight): Control series | Liver weight: Tumour-bearing series | Adrenal weight (mg./100 g. of initial body weight): Control series | Adrenal weight: Tumour-bearing series | Spleen weight (mg./100 g. of initial body weight): Control series | Spleen weight: Tumour-bearing series |
|---|---|---|---|---|---|---|---|---|
| None | Low | 11·4±2·8 | 2·78±0·30 | 3·14±0·34 | 31·4±1·4 | 41·0±4·9 | 348±62 | 426±84 |
| | High | 9·3±2·2 | 2·81±0·15 | 3·61±0·13 | 29·4±4·3 | 39·8±4·6 | 353±41 | 553±102 |
| Adequate | Low | 10·5±2·0 | 2·64±0·14 | 3·91±0·35 | 31·0±3·6 | 48·2±6·1 | 393±70 | 821±142 |
| | High | 9·3±2·4 | 3·20±0·15 | 4·22±0·68 | 32·0±2·0 | 49·4±8·7 | 374±12 | 813±133 |

with the observation of Green *et al.* (1950) who found that growth of the Walker 256 carcinoma was sensitive to the nutritional state of the host only during the first 5–6 days after implantation.

The liver, spleen and adrenals of tumour-bearing rats are larger than those of normal rats (Stewart and Begg, 1953). In the present work increases in the weights of these organs were observed under all dietary conditions (Table I). The gain in liver weight caused by the tumour was not significantly influenced by diet. The increases in spleen and adrenal weights in tumour-bearing animals did appear to be more marked in the animals receiving a protein-containing diet ($P < 0{\cdot}05$).

*The effect of the Walker 256 carcinoma on the protein and nucleic acid content of rat liver*

Stewart and Begg (1953) found that the Walker 256 carcinoma produced liver protein deposition in rats fed a high protein diet. In the present study an increase in liver protein content was observed in tumour-bearing rats. irrespective of the level of protein and energy fed (Table II). This increase in liver protein was

TABLE II.—*A Comparison of the Protein N, RNAP and DNAP Content of the Livers of Tumour-bearing and Control Rats under Various Dietary Conditions*

Results are the means of 6 experiments ± S.E. Analysis of variance shows that the tumour caused significant increases in liver protein, RNA and DNA content ($P < 0{\cdot}01$ in all cases) and that these effects are independent of dietary protein and calorie intake ($P > 0{\cdot}05$ for all interactions).

| | | Liver protein N (mg./100 g. of initial body weight) | | | Liver RNAP (mg./100 g. of initial body weight) | | | Liver DNAP (mg./100 g. of initial body weight) | | |
|---|---|---|---|---|---|---|---|---|---|---|
| Dietary protein | Dietary calorie value | Control series | Tumour-bearing series | Mean percentage difference | Control series | Tumour-bearing series | Mean percentage difference | Control series | Tumour-bearing series | Mean percentage difference |
| None | Low | 57·3±5·6 | 65·1±5·4 | +14 | 2·71±0·19 | 3·64±0·14 | +34 | 1·07±0·11 | 1·18±0·08 | +10 |
| | High | 53·6±6·6 | 68·5±5·7 | +28 | 2·63±0·14 | 3·90±0·34 | +52 | 1·04±0·09 | 1·19±0·02 | +16 |
| Adequate | Low | 57·4±6·2 | 85·9±9·4 | +50 | 2·68±0·23 | 4·37±0·51 | +63 | 1·08±0·10 | 1·29±0·07 | +19 |
| | High | 72·0±8·1 | 86·9±17·9 | +21 | 3·07±0·22 | 4·49±0·89 | +46 | 1·09±0·10 | 1·29±0·11 | +18 |

also accompanied by increases in both liver RNA and DNA, protein and calories intake again having no significant effect on the magnitude of these changes (Table II).

In each dietary group the magnitude of the increase in liver RNA content (34–63 per cent) was much greater than that in either liver protein (14–50 per cent) or DNA (10–19 per cent).

*Comparison of the intracellular distribution of RNA and protein in the livers of tumour-bearing and control rats*

Since the Walker 256 carcinoma produced an increase in both liver RNA and protein content a study of the intracellular distribution of RNA and protein in the livers of tumour-bearing and control rats was undertaken in an attempt to ascertain whether these increases were due to changes in any particular fractions of the liver cell. In the preceding series of experiments it was found that the increases in RNA and protein were independent of the dietary state of the animal.

Consequently in the present study only tumour-bearing and control rats receiving an adequate protein and calorie intake were employed. Table III indicates that, although there was a considerable increase in the amount of RNA in the livers of tumour-bearing aninals (P < 0·1), the percentage distribution of RNA in the different subcellular fractions was not significantly different from that found in the normal rat liver.

TABLE III.—*The Intracellular Distribution of RNAP and Protein N in the Livers of Control and Tumour-bearing Rats*

The results are the means of 4 experiments ± S.E. in the case of RNAP and 3 experiments ± S.E. in the case of protein N. Statistical analysis (" t " test) shows that the tumour caused a significant increase in total liver RNA content (P < 0·01); that there was no significant alteration in the pattern of RNA distribution in the subcellular fractions of the livers of tumour-bearing rats; that there was a significant fall in the proportion of protein N present in the mitochondrial fraction of the livers of tumour-bearing animals (P < 0·05).

| Treatment | Total amount per liver (mg./100 g. of initial body weight) | Distribution in the subcellular fractions (expressed as a percentage of the whole homogenate) | | | | |
|---|---|---|---|---|---|---|
| | | Nuclear | Mito-chondrial | Micro-somal | Soluble | Recovery |
| RNAP— Control | 3·01±0·03 | 7·6±0·9 | 13·5±1·4 | 49·6±2·0 | 25·1±1·9 | 95·8±2·3 |
| Tumour-bearing | 5·64±0·61 | 9·1±0·9 | 11·5±3·3 | 45·7±2·8 | 28·7±5·5 | 95·0±2·8 |
| Protein N— Control | 74·5±0·7 | 15·9±0·3 | 28·6±2·8 | 18·6±3·0 | 35·6±3·4 | 98·6±6·5 |
| Tumour-bearing | 88·3±6·7 | 20·5±3·8 | 13·7±3·6 | 19·9±3·3 | 39·9±4·4 | 94·0±1·8 |

The protein data (Table III) suggest that there was again an increase in the protein content of the livers of tumour-bearing animals. However due to the variation between animals this increase did not attain statistical significance. Nevertheless the pattern of distribution of protein in the different subcellular fractions was significantly altered by the presence of a tumour, namely by a fall in the proportion of the cellular protein present in the mitochondrial fraction (P < 0·05).

*Mean DNA content of nuclei isolated from the livers of control and tumour-bearing rats*

Since the total DNA content of the livers of tumour-bearing rats was increased, a study was made to determine whether this rise was accompanied by an increase in the mean nuclear DNAP. The dietary conditions were similar to those described in the preceding section. DNAP was estimated both by phosphorus determination following the Schmidt and Thannhauser procedure (1945) for the isolation of DNA and by the diphenylamine method described by Schneider (1957).

Table IV shows that although the total DNA content of the liver is considerably increased in tumour-bearing animals (P < 0·01) the mean DNA content per liver nucleus did not differ from that obtained in normal rats.

TABLE IV.—*A Comparison of the Mean DNAP Content of Liver Cell Nuclei in Control and Tumour-bearing Rats*

Results are the means of 7 experiments $\pm$ S.E. Statistical analysis (" t " test) shows that there was a significant increase in the DNAP content of livers of tumour-bearing rats ($P < 0{\cdot}01$) but no alteration in the mean DNAP content of the nucleus.

| Treatment | Tumour weight (g.) | Liver DNAP (mg./100 g. of initial body weight) | DNAP (pg./nucleus) as determined by Phosphorus estimation | Deoxypentose estimation |
|---|---|---|---|---|
| None | — | 0·97±0·02 | 0·96±0·02 | 0·90±0·03 |
| Tumour | 9·9±0·8 | 1·20±0·06 | 0·95±0·04 | 0·90±0·03 |

*Histological examination of the livers of control and tumour-bearing rats*

Histological examination of the livers of tumour-bearing rats failed to show any evidence of centres of haematopoiesis such as have been described by Ventura, Richer and Selye (1957) in livers of rats bearing large Walker tumours (ca. 40 g.).

### DISCUSSION

In the present series of experiments it has been found that the Walker 256 carcinoma causes an increase in the weights of the liver, spleen and adrenal glands of the host. The increase in liver size was independent of the level of protein and calorific value of the diet while the increases in spleen and adrenal weights were more marked in rats receiving a protein-containing diet. Tumour growth also appeared to be independent of the dietary conditions employed.

Examination of liver composition showed that there was an increase in the total amounts of protein, RNA and DNA in the livers of tumour-bearing rats, irrespective of the protein and calorie content of the diet. The fact that even the livers of tumour-bearing rats receiving a protein-free diet showed this increase suggests that the protein laid down in the liver arises from the breakdown of carcass protein which is known to occur in tumour-bearing animals (Sherman, Morton and Mider, 1950 ; Begg and Dickinson, 1951). It is of interest to note that Green *et al.* (1950) could not demonstrate any increase in the protein content of the livers of rats bearing the Walker 256 carcinoma when these animals had been extensively depleted of body protein by feeding them a protein-free diet for three weeks before tumour inoculation.

This action of the tumour in causing an increase in liver protein by depleting the carcass appears similar to the action of cortisone. There is evidence that this hormone acts primarily on the carcass causing a breakdown of tissue protein resulting in an increased supply of amino acids to the liver (Goodlad and Munro, 1959). Due to the sensitivity of the growth of the Walker 256 carcinoma to dietary protein (Green *et al.*, 1950) the dietary conditions employed in the present work were not exactly similar to those of a preceding study of the action of diet on the response of the liver to cortisone adminstration (Goodlad and Munro, 1959). Nevertheless there would appear to be certain differences in the response of the liver to these two circumstances which are not related to the change in experimental conditions. In the first place, it was found that the deposition of liver protein and RNA caused by cortisone was related to the calorie intake, whereas the increase of these two constituents in the livers of tumour-bearing rats did not vary with calorie intake. Secondly, although cortisone was without effect on liver DNA, the tumour caused a significant rise in the total content of DNA in the liver. Finally, the relative increase in liver RNA with respect to the increase in liver protein was much greater in the tumour-bearing rats than in the cortisone-treated rats. These results therefore suggest that the changes observed in liver composition in tumour-bearing animals may be brought about by a different mechanism from that by which cortisone exerts its effect.

Studies on the intracellular distribution of RNA and protein showed that, while the RNA of all subcellular fractions was increased by the action of the tumour, the percentage of the total liver protein present in the mitochondrial fraction was lowered in tumour-bearing animals. Whether this was due to a decrease in the amount of protein per mitochondrion or to a decrease in the number of mitchondria relative to the other cellular components cannot be decided from the data available. Allard, de Lamirande and Cantero (1953) have in fact found that the mitochondrial population (expressed as mitochondria per cell) of liver cells adjacent to a growing hepatoma but themselves free of tumour was significantly lower than that of normal liver cells.

The total DNA content of the livers of tumour-bearing rats was significantly increased. A rise in liver DNA may be attributed to one of the following :—

(*a*) Centres of extramedullary haematopoiesis may be present. These have been observed in the liver and other organs of tumour-bearing animals (Begg, 1958).

(*b*) The degree of polyploidy of liver nuclei may have increased. Leuchtenberger, Leuchtenberger and Myeki (1958), working with mice, showed that the amount of DNA per liver nucleus increased folloing injection of tumour DNA.

(*c*) There may be formation of new liver nuclei.

Thomson, Heagy, Hutchison and Davidson (1953) have investigated the mean DNA content per nucleus of cells isolated from different tissues of the rat. They found that, while the mean DNAP content of liver nuclei was of the order of 0·90 pg. per nucleus or greater, that of spleen and bone marrow tissues, where active haematopoiesis occurs, was only 0·63 pg. and 0·67 pg. per nucleus respectively. Leucocyte nuclei had a mean DNAP content of 0·64 pg. per nucleus. Thus a fall in the mean DNA content of liver cell nuclei might be expected if infiltration of haematopoietic centres was occurring to any great extent. An increase in polyploidy, on the other hand, would be reflected by an increase in the mean nuclear DNA content. The fact that the DNA content of liver cell nuclei was not altered and the absence of any histological evidence of haematopoietic centres suggest that the rise in total liver DNA in tumour-bearing rats is due mainly to the formation of new nuclei. This view receives further support from the report that resting liver mitosis rates in rats bearing the Walker 256 carcinoma were seven times greater than normal (Hemingway, 1960).

### SUMMARY

1. The effect of the Walker 256 carcinoma on the tissues of the host has been studied in rats receiving diets varying in protein and calorie content.

2. Tumour growth was found to be independent of the various dietary treatments employed.

3. The liver, spleen and adrenals of tumour-bearing rats were larger than those of normal rats. This increase in weight was independent of the level of protein and calorie content of the diet in the case of liver but the rises in spleen and adrenal weights observed in tumour-bearing animals were more marked in rats receiving a protein-containing diet.

4. An increase in the protein, RNA and DNA content in the liver was observed in tumour-bearing rats, under all dietary conditions. The percentage increase in RNA being much greater than that of either protein or DNA.

5. The intracellular distribution of RNA in liver was unaffected by the presence of a tumour. However protein distribution was altered in the livers of tumour-bearing rats, there being a fall in the proportion of protein found in the mitochondrial fraction.

6. The mean DNA content of liver nuclei in rats bearing the Walker carcinoma did not differ from that observed in normal rat liver.

The authors wish to express their gratitude to Dr. H. N. Munro of the Department of Biochemistry, Glasgow University, for valuable advice and discussion, Professor A. Haddow F.R.S. of the Chester Beatty Research Institute, London, for supplying a suitable strain of rats and samples of the Walker 256 carcinoma and Dr. W. W. Park of the Department of Pathology, St. Andrew's University, for advice on certain histological points. One of the authors (G. A. J. G.) carried out part of this work during the tenure of a Beit Memorial Fellowship at the Department of Biochemistry, Glasgow University, and would like to thank Professor J. N. Davidson, F.R.S., for facilities granted. The latter stages of this work were supported by a grant from the Medical Research Council for expenses and scientific assistance to one of us (G. A. J. G.) for which we are most grateful.

### REFERENCES

Allard, C., de Lamirande, G. and Cantero, A.—(1953) *Canad. J. med. Sci.*, **31**, 103.

Allison, J. B., Bernstein, E. H. and Babson, A. L.—(1954) *Fed. Proc.*, **13**, 174.

Begg, R. W.—(1958) *Advanc. Cancer Res.*, **5**, 1.

*Idem*, and Dickinson, T. E.—(1951) *Cancer Res.*, **11**, 409.

Goodlad, G. A. J. and Munro, H. N.—(1959) *Biochem. J.*, **73**, 343.

Green, J. W., Benditt, E. P. and Humphreys, E. M.—(1950) *Cancer Res.*, **10**, **769**.

Hemingway, J. T.—(1960) *Nature, Lond.*, **185**, 106.

Leuchtenberger, C., Leuchtenberger, R. and Myeki, E.—(1958) *Proc. nat. Acad. Sci., Wash.* **44**, 700.

Munro, H. N.—(1949) *J. Nutr.*, **39**, 375.

*Idem* and Naismith, D. J.—(1953) *Biochem. J.*, **54**, 191.

Schmidt, G. and Thannhauser, S. J.—(1945) *J. biol. Chem.*, **161**, 83.

Schneider, W. C.—(1948) *Ibid.*, **176**, 259.

*Idem*—(1957) in " Methods in Enzymology ", vol. 3, p. 680, Ed. by Colowick, S. P. and Kaplan, N. O., New York (Acad. Press Inc.).

Sherman, C. D., Morton, J. J. and Mider, G. B.—(1950) *Cancer Res.*, **10**, 374.

Silber, R. H. and Porter, C. C.—(1953) *Endocrinology*, **52**, 518.

Smellie, R. M. S., Humphrey, G. F., Kay, E. R. M. and Davidson, J. N.—(1955) *Biochem. J.*, **60**, 177.

Stewart, A. G. and Begg, R. W.—(1953) *Cancer Res.*, **13**, 556, 560.

Talalay, P., Takano, G. M. V. and Huggins, C.—(1952) *Ibid.*, **12**, 834.

Thomson, R. Y., Heagy, F. C., Hutchison, W. C. and Davidson, J. N.—(1953) *Biochem. J.*, **53**, 460.

Trémolières, J., Derache, R. and Lowy, R.—(1955) *Ann. Nutr., Paris*, **9**, 179.

Ventura, J., Richer, C-L. and Selye, H.—(1957) *Cancer Res.* **17**, 215.